# QoS Improvement Using In-Network Caching Based on Clustering and Popularity Heuristics in CCN

**DOI:** 10.3390/s21217204

**Published:** 2021-10-29

**Authors:** Sumit Kumar, Rajeev Tiwari, Wei-Chiang Hong

**Affiliations:** 1 Department of Systemics, School of Computer Science, University of Petroleum and Energy Studies, Bidholi, via Prem Nagar, Dehradun 248007, India; Sumit.kumar@ddn.upes.ac.in; 2 Department of Systemics, University of Petroleum and Energy Studies, Bidholi, via Prem Nagar, Dehradun 248007, India; rajeev.tiwari@ddn.upes.ac.in; 3 Department of Information Management, Asia Eastern University of Science and Technology, New Taipei 22064, Taiwan

**Keywords:** content-centric networking, content caching, network clustering, content popularity

## Abstract

Content-Centric Networking (CCN) has emerged as a potential Internet architecture that supports name-based content retrieval mechanism in contrast to the current host location-oriented IP architecture. The in-network caching capability of CCN ensures higher content availability, lesser network delay, and leads to server load reduction. It was observed that caching the contents on each intermediate node does not use the network resources efficiently. Hence, efficient content caching decisions are crucial to improve the Quality-of-Service (QoS) for the end-user devices and improved network performance. Towards this, a novel content caching scheme is proposed in this paper. The proposed scheme first clusters the network nodes based on the hop count and bandwidth parameters to reduce content redundancy and caching operations. Then, the scheme takes content placement decisions using the cluster information, content popularity, and the hop count parameters, where the caching probability improves as the content traversed toward the requester. Hence, using the proposed heuristics, the popular contents are placed near the edges of the network to achieve a high cache hit ratio. Once the cache becomes full, the scheme implements Least-Frequently-Used (LFU) replacement scheme to substitute the least accessed content in the network routers. Extensive simulations are conducted and the performance of the proposed scheme is investigated under different network parameters that demonstrate the superiority of the proposed strategy *w.r.t* the peer competing strategies.

## 1. Introduction

The Internet is initially designed as a “collection of hosts” which is used to access available resources that are distributed in the network. The traditional TCP/IP Internet architecture supports the host-centric content retrieval mechanism, where the contents are accessed using the IP addresses of network nodes. The Internet has become a global infrastructure and with its tremendous growth in applications, the IP-based network traffic is estimated to be 4712 Exabytes per year at the end of 2022 [1]. Moreover, modern Internet applications [2,3] impose intensive Quality-of-Service (QoS) requirements during content retrieval operations such as minimal content access delay, network traffic, and effective use of available network resources, etc. The quality improvements in the IP-based environment have various techniques implied in recent research as per authors Tiwari et al. [4,5]. However, the patch-based TCP/IP architecture starts showing its limitations towards the current Internet applications and their increased new requirements due to its host-centric nature [6,7].

In this context, the Content-Centric Networking (CCN) is proposed as a clean slate architecture for the future Internet [8]. CCN supports a content-name-based data retrieval mechanism instead of searching for the IP address-based host in the network to access the required data. Thus, the data can be retrieved from any network node that has a copy of the requested content in CCN. Furthermore, the CCN offers the in-network caching capability and the requested contents can be served from the origin servers or the cache of nearby intermediate network routers. The underlying content caching improves QoS for the end-users by minimizing content retrieval delay, reducing the load on the network nodes, and traffic during data dissemination [9,10].

The in-network content caching policy takes decisions related to the selection of suitable locations for the content placement and selection of older contents for replacement operations when the cache becomes full. These caching policies are generally categorized into on-path and off-path caching schemes [11]. In on-path schemes [12], the content is cached in the intermediary routers that forward the content from the content provider towards the requester. In recent, several on-path caching schemes are proposed by various researchers that takes content placement decisions based on the content popularity [13,14], node importance [10,15], content age [16], and distance-based parameters [17,18], etc. Contrarily, the off-path schemes can place the content in any of the network router that may or may not exist in the content delivery route. Generally, the off-path caching schemes considers a hash-based mechanism during content caching decisions such as [19,20,21]. Due to hash-based content caching decisions, most of the off-path caching schemes suffers from higher network traffic and increased path stretch. Additionally, these schemes do not consider the content popularity or topological information during content placement decisions. In contrast to these schemes, the on-path schemes creates lesser communication overhead and computational complexity during content caching decisions. Therefore, the on-path caching schemes are widely implemented in the CCN. After exhaustive analysis of the existing on-path caching strategies, there are mainly two reasons that motivated us for the proposed content caching scheme.

- *Network traffic and redundancy:* The conventional on-path caching policy of CCN, called ubiquitous caching [22] allows each intermediary router in the retrieval path to temporarily store the incoming contents. This increases the availability of contents near the end-user devices and reduces content retrieval delay up to certain extent. However, the scheme suffers from higher content redundancy as the same content is placed in all the on-path routers during content forwarding. Due to this, the other content requests need to be served by the server, which causes excessive network traffic due to poor cache diversity. This leads to degraded network performance and QoS for end user devices. Therefore, although caching of contents in the intermediate routers improves network performance, the determination of appropriate network routers and the selection of contents for the caching operations is an open research gap that needs to be addressed.
- *Content retrieval delay:* Most of the existing on-path caching schemes takes autonomous caching decisions. Before forwarding the content to downstream nodes, each on-path router needs to perform certain computations for content caching decisions. This excessive computation for content caching becomes an obstruction in real-time content delivery and also causes excessive consumption of computational resources in the network routers. Therefore, it is essential to reduce the computational delay during caching decisions and the suitable contents need to be placed in appropriate network routers.

With these motivations, the objective of this paper is to propose an efficient content caching scheme that reduces the content retrieval delay and resource consumptions to offer improved network performance in CCN networks. Towards this, the proposed scheme provides two-folded content caching strategy. First, it partitioned the network nodes into the non-overlapping clusters using the topological information of the network. The clustering is performed to reduce content placement/replacement operations and to decrease computational latency in the network routers. During content retrieval, at most one copy of the incoming content is cached in that cluster from where the request is generated. The intermediate routers that do not belong to requester’s cluster in the path, cannot cache the forwarded contents. Hence, the computational latency is significantly reduced for the network routers. Secondly, to take caching decisions, the proposed scheme considers the content popularity and the hop count information to place popular contents near the end-user devices. When an intra-cluster router cache the incoming content, the remaining routers of that cluster just forward the content towards the requester without further caching operations. Thus, the proposed heuristics also control the excessive content redundancy and lead to comprehensive use of the caching capacities of the network. The major contributions of the paper are as follows:

- A clustering-based in-network content caching scheme is proposed for the CCN to improve QoS for end-user devices and comprehensive use of cache space. By clustering the network nodes, the proposed scheme constrains excessive caching operations and content redundancy in the network.
- The proposed caching scheme considers content popularity and hop-count metrics along with the clusters information for the caching decisions. Using these heuristics, the caching probability increases for the frequently accessed contents near the end-user devices to reduce content access delay.
- The performance of the proposed caching scheme is examined through extensive simulations on the realistic network topology. Simulations results show the necessity of the proposed clustering-based caching scheme since the conventional scheme does not achieve a considerable hit rate in the network. Moreover, the proposed scheme demonstrates a significant decrease in the content retrieval delay and network traffic from the existing caching strategies.

The organization of the remaining paper is as follows. The next section (Section 2) provides the overview of CCN. Section 3 discuss the brief survey of the prior related works. The system model is presented in Section 4. In Section 5, the novel clustering and the caching schemes are proposed. The performance of the proposed scheme is evaluated and compared with peer caching schemes in Section 6. Finally, the paper is concluded in Section 7.

## 2. Overview of CCN Architecture

This section briefly describes the CCN architecture and its operations to provide the foundation for further discussions. As CCN is a data-centric network, the content retrieval mechanism relies on two types of messages: Interest message and Content message [23]. The end-user device generates the Interest message to request for the specific content and the in-network router/provider replies with the corresponding Content message. For the routing and caching operations, each router maintains a Forwarding Information Base (FIB), Content Store (CS) and the Pending Interest Table (PIT) [24]. The FIB contains the interface information to forward the Interest message towards the content source. The incoming content can be cached in the CS of on-path routers based on the caching policy. When a router receives an Interest message from one or more interfaces, the information of those pending Interest messages and their interfaces is stored in the PIT.

On receiving the Interest message from the end-user device, the network router first searches its CS for the requested content. If a cache hit occurs then the Content message is created by the router and forwarded towards the end-user device using the interface through which the Interest message arrived. If a cache miss occurs, then the router investigates its PIT. If a matching entry is found in PIT then the interface information of the incoming Interest message is aggregated in the PIT and the message is disposed from the network. Otherwise, a record is created in the PIT and the Interest message is forwarded towards the source using FIB.

When an intermediate router receives a Content message, it checks its PIT for the matching records. If the entry is found then the router forwards the Content message toward those interfaces that are mentioned in the PIT and cache the Content message in its CS based on the content placement and replacement policies. After content forwarding, the router removes entries for that Content message from the PIT.

## 3. Literature Review

In-network content caching is an inherent characteristic of CCN architecture that raises several challenges during content placement and replacement operations. To improve the network performance and QoS for the end-user devices, various content caching schemes are proposed by the research community [25,26]. The traditional Leave-Copy-Everywhere (LCE) [27] caching scheme places the content in each intermediate router throughout the delivery path. The scheme cache the contents near the end-user devices and reduces content retrieval delay for future Interest messages. However, this excessive caching causes high energy consumption and cache replacement operations. Moreover, the excessive content redundancy also increases cache miss probability as the cache size is limited in realistic networks. Therefore, a trade-off exists between the caching and no-caching operations. Excessive caching operations can reduce the latency up to a certain extent but causes extreme exploitation of network resources. On the other side, no-caching in the network routers leads to higher delays and network traffic. Hence, it is necessary to focus on frequently requested contents and suitable locations for optimal network performance.

For content placement decisions, a random probability-based caching scheme called RandProb is proposed in [28]. The scheme randomly places the incoming contents in the on-path routers and does not involve significant computational latency during caching decisions. To reduce cache replacements, the Leave-Copy-Down (LCD) scheme is suggested in [29] that drops the accessed content one-hop downside from the content provider. With this, the frequently accessed contents are gradually placed towards the edges of the network. The Probcache caching strategy [18] approximates the caching capacity of the path and multiplex the contents between the server and the end-user device (requester). Using the proposed mechanism, the Probcache scheme fairly allocates the network resources among different network flows. However, these caching schemes [18,27,28,29] do not consider the router’s characteristics and content popularity during caching decisions and hence unable to make efficient use of caching resources.

To increase cache hit probability on those routers that observe high network traffic, various centrality-based caching schemes are also proposed [30]. A betweenness centrality-based caching approach is suggested in [31] that eliminates the uncertainty of random-probability-based content placement decisions and shows improved caching gains. An in-depth comparison of several centrality-metrics-based caching mechanisms has been performed in [15] that involve Degree Centrality (DC-based), Stress Centrality, Betweenness Centrality, etc. The results illustrate that the degree centrality is a simple and effective parameter for efficient cache use. The CPNDD (Content Placement based on Normalized Node Degree and Distance) caching scheme [17] shows that considering a single parameter for the caching decisions does not achieve significant performance gain. The scheme suggests to jointly consider the degree centrality and hop count parameters for content placement decisions. Using these parameters, the caching probability increases in those routers that have a high degree centrality and are far from the content provider. The results show improved cache hit ratio and reduction in server load from LCE and DC-based caching strategies.

Various researchers have also recommended considering the content popularity for caching decisions in the network. Towards this, in the Most-Popular Content Caching (MPC) scheme [32], each router computes content access frequencies autonomously. When the content becomes popular enough, the router suggests its adjacent routers to cache the popular content in their storage. Using this approach, the cache redundancy increases for popular contents in the network. The Content Popularity and User Location (CPUL)-based caching scheme [33] divides the contents into popular and normal contents using a centralized server. The scheme then suggests taking caching decisions based on the type of content and the user location in the network. However, as defined in the scheme, the determination of content popularity on a centralized server causes scalability concerns for large-scale networks. The Dynamic Popularity Window-based Caching Scheme (DPWCS) [14] proposed to implement a large popularity window in each network router, which is used to determine the popularity of contents. The scheme identifies popular contents based on the request distribution model, caching capacity of the routers, and the number of distinct contents in the networks. One of our prior work proposed in Tiwari et al. [34] discusses a content Popularity and Distance-based Caching scheme (PDC) for content placement/replacement decisions. The scheme jointly considers the content popularity and hop count-based distance attributes during content caching in the network and shows improved network performance as compared to conventional LCE and DC-based caching strategies.

However, most of the above discussed caching schemes [14,15,17,27,28,29,34] take autonomous caching decisions where routers do not cooperate for content placement operations. Although autonomous content caching reduces communication overhead in the network, these scheme suffers from higher content redundancy and cache replacement operations. Moreover, many schemes consider at most one parameter for the caching decisions such as node centrality, content popularity, and hop count [18,29,30,31,32]. Due to this, these schemes suffers from load imbalance events as the routers that are near the server or have a higher degree centrality would experience more caching operations as compared to other routers in the network.

To alleviate the load im-balancing issues and reduction in excessive caching operations, several cluster-based caching schemes are also proposed in the CCN [35,36,37,38]. The Hierarchical Cluster-based Caching (HCC) scheme [35] partitioned the network routers into the core routers and the edge routers. The core routers do not have caching capability and the few selected edge routers can cache the contents. For caching decisions, the scheme jointly considers node degree centrality, hop-count, and delay metrics. In [36], the authors proposed k-split and k-medoid clustering schemes to partition the network. The scheme performs hash-based caching operations and thus, it does not consider content or router’s characteristics during content placement decisions. The scheme mentioned in [37] creates a fixed number of partitions in the network based on the hop count information. The scheme performs caching operations using the partition information and the content popularity in the network. A cluster-based scalable scheme is suggested in [38] that combines the physical routers together and these routers are seen as a single unit to the outside nodes. However, internally, the traffic load has been distributed among the physical routers.

Once the cache of the network routers becomes full, the older content needs to be evicted to cache the incoming content. Generally, this cache replacement operation is performed using the First-In-First-Out (FIFO), Least-recently Used (LRU), Least-Frequently-used (LFU), and optimal cache replacement strategies [39,40]. As discussed in [39,41], the optimal replacement scheme achieves improved network performance as compared to peer schemes. However, the implementation of the optimal strategy is not feasible as the content requests pattern cannot be predicted in realistic network topologies. Due to this, the LRU and LFU algorithms are widely implemented with the content placement schemes due to their sensitivity towards content access pattern and content popularity, respectively.

The distinguishing features of the reviewed caching strategies are summarized in Table 1. As defined in Table 1, in most of the existing on-path caching schemes the routers take caching decisions independently and do not cooperate with each other. This leads to excessive number of caching operations and increases duplicate contents in the network. Due to this, the existing schemes achieves limited gain in the network performance. Additionally, the existing clustering-based caching schemes have not explored the joint effect of content popularity and the distance attributes on caching performance.

Therefore, a novel network clustering scheme is proposed in this paper for efficient use of the caching resources and improved QoS for the end-users. The proposed scheme considers hop-count and link bandwidth information to form tightly coupled clusters. Then, the proposed caching scheme jointly considers the clustering information, content popularity, and the content provider distance for caching decisions. With this, the popular contents are placed near the end-users with fairly multiplexed content redundancy in the path. This makes the proposed scheme suitable for CCN-based applications.

## 4. System Model and Assumptions

**Figure 1:**
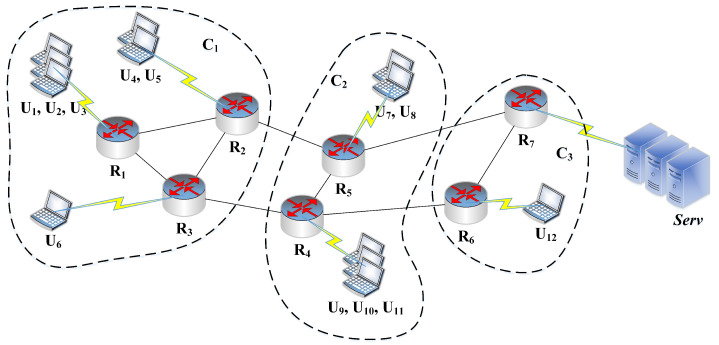
An illustration of network clustering and the caching strategy.

Let G(V,E) be a network topology having a set of nodes represented as *V* = {$U_{1}$, $U_{2}$, …, $U_{\left|u\right|}$, $R_{1}$, $R_{2}$, …, $R_{\left|r\right|}$, serv}. Here, *E* denotes the set of connections that are used for the Interest/Content message forwarding among nodes in the network. Figure 1 illustrates an example of the network topology. Here, $U_{i}$ represents the *i*th end-user device and it generates Interest messages in the network. The $R_{i}$ denotes *i*th router in the network and these routers perform Interest/Content message forwarding and caching operations. The notation (serv) defines the servers in the network and each server works as an Interest message sink that satisfies all Interest messages. In the system, all the network routers have caching capability (for simplicity, although it is not necessary) and the decisions related to content placement depend on several parameters as described in Section 5. Our recent studies [14,34] establish the effective heuristics for the determination of content popularity that can assist in computing the content access frequencies. However, these previously suggested schemes take autonomous caching decisions and have a further scope of improvement using cooperation among network nodes.

To simplify further discussions, the notations used in the model are defined in Table 2. It has been assumed that the content packets are of fixed size and the content access pattern follows Zipf distribution model [15,42]. The Zipf distribution is widely implemented in large-scale networks to model realistic network traffic patterns as it assigns ranks to the contents based on their popularity. Here, content popularity is defined as the content access frequency from the catalogue [10]. It has also been assumed that the proposed scheme implements a request-response model [43] of Content-Centric Networking. In this model, the Content message follows the same route through which the Interest message arrived at the content provider. In general, these assumptions are unbiased under consideration of location independence and name-based routing features of CCN.

As shown in Figure 1, the network has been partitioned into three clusters namely $C_{1},\;C_{2},$ and $C_{3}$ using the proposed network clustering scheme elaborated in the subsequent section. Cluster $C_{1}$ contains routers $R_{1}$, $R_{2}$ and $R_{3}$ and the end-user devices $U_{1}$ to $U_{6}$. In other words, $\left\{R_{1},R_{2},R_{3},U_{1},U_{2},\ldots ,U_{6}\right\}\in C_{1}$. Similarly, $\left\{R_{4},R_{5},U_{6},\ldots U_{11}\right\}\in C_{2}$ and $\left\{R_{6},R_{7},U_{12}\right\}\in C_{3}$. Suppose, the end-user device $U_{3}$ generates an Interest message for the content name “\prefix\xyz” and forward this message towards the server. Lets assume that the Interest message follows a path $U_{3}\to R_{1}\to R_{2}\to R_{5}\to R_{7}\to Serv$ and no intermediate router have a copy of the requested content. Then, the server would prepare the corresponding Content message with the required payload and transmit it in the backward direction towards $U_{3}$. In the proposed caching scheme, at most one copy of the incoming content would be cached in the cluster from where its request is generated $\left(C_{1};\;as\;U_{3}\in C_{1}\right)$. As the Interest message for content “\prefix\xyz” is generated from $U_{3}\in C_{1}$, the on-path routers $R_{1}\;and\;R_{2}$ would take content placement decisions based on the content popularity and the hop count parameters (discussed in Section 5.5). Thus, the remaining intermediate routers in the path $(R_{5}$ and $R_{7})$ simply forward the content “\prefix\xyz” towards $U_{3}$ without caching operation as $\left\{R_{5},R_{7}\right\}\notin C_{1}$. Therefore, the content redundancy and the number of caching operations are reduced significantly in the network. It has been argued that this would lead to lower content retrieval delay, network traffic, and improved QoS for the end-user devices.

For caching decisions, the content popularity and hop count metrics are determined using the following concepts:

**Content popularity determination using Popularity Table**: According to the Zipf distribution, there are always few content requests for the unpopular contents in the network. If the caching scheme does not consider content access patterns during placement decisions, then the unpopular contents may be stored for longer durations in the network routers without being accessed again. This leads to poor use of network resources as cache miss probability increases due to caching of unpopular contents. Moreover, it has also been observed that the few routers with high importance receive more number of Interest messages as compared to other routers in the network. To resolve these issues, our previous work [17] has suggested to integrate a large size Popularity Table with each network router. This table is used to determine the content access frequency. The Popularity Table stores only the name of the requested content in its slots $\left(PT_{R_{i}}^{s}\right)$ and hence, this has negligible space overhead on the routers. When, the Popularity Table reaches its maximum size $(Max(|PT_{R_{i}}\left|)\right)$, then First-In-First-Out (FIFO) replacement mechanism is used to evict oldest content request from the table to store incoming request information. During caching decisions, the router computes the popularity of the incoming content by counting its occurrences in the Popularity Table.

**Figure 2:**
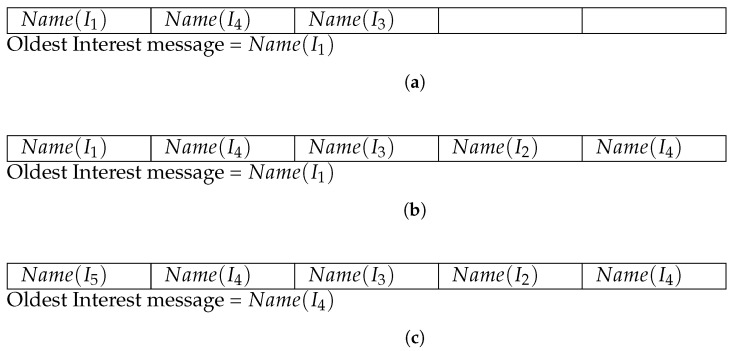
An illustration of the management of Interest message information in the Popularity Table. (**a**) $Max(|PT_{R_{i}}|)=5$, $|PT_{R_{i}}|=3$; (**b**) $Max(|PT_{R_{i}}|)=5$, $|PT_{R_{i}}|=5$ (after arrival of $I_{2}$ and $I_{4}$); (**c**) $Max(|PT_{R_{i}}|)=5$, $|PT_{R_{i}}|=5$ (after arrival of $I_{5}$).

Figure 2 illustrates the working of the Popularity Table. Suppose, the maximum size of the Popularity Table $Max(|PT_{R_{i}}|)$ is 5. Figure 2a shows the structure of a Popularity Table, implemented in a specific router $\left(R_{i}\right)$, after arrival of Interest messages: $I_{1}$, $I_{4}$, and $I_{3}$ in a sequence. As shown in the figure, only the name of the requested contents $\left(Name\left(I_{i}\right)\right)$ are stored in the Popularity Table and therefore, this structure does not causes significant storage overhead in the cache. In Figure 2a, two slots of the Popularity Table are empty and it has been described as $Max(|PT_{R_{i}}|)=5$ and $|PT_{R_{i}}|=3$. After arrival of Interest message $I_{2}$ and $I_{4}$, the empty slots of the Popularity Table are updated as demonstrated in Figure 2b and the structure reaches to its maximum capacity $(Max(|PT_{R_{i}}|)=|PT_{R_{i}}\left|=5\right)$. When a new Interest message $\left(I_{5}\right)$ arrives, the router determines that the Popularity Table has no free slot and hence, the FIFO replacement algorithm is used to evict the oldest content name from the Popularity Table to store the information of incoming Interest message. Therefore, the information of oldest Interest message $\left(I_{1}\right)$ is replaced with $Name(I_{5})$ as shown in Figure 2c and now, $Name(I_{4})$ becomes the oldest content (slot-2) for eviction during future Interest message arrival.

**Hop count monitoring**: The hop count is a simple and effective metric to increase caching probability towards the edges of the network [18,34]. The hop count metric for the Interest/Content message has been computed as the number of hops (routers/server) traversed by the message to reach the content provider/requester, respectively.

## 5. Proposed Caching Scheme

In this section, the proposed network clustering scheme is discussed in Section 5.1. Section 5.2 defines the updated structures of the Interest and Content message for the caching decisions. Then, the proposed Interest and Content message processing mechanisms are introduced in Section 5.3 and Section 5.4, respectively.

### 5.1. Proposed Clustering Scheme

Algorithm 1 shows the proposed clustering mechanism to form the clusters. The intra-cluster nodes collaborate with each other to take caching decisions without any additional communication overhead. In the proposed clustering strategy, initially the top “k” routers are identified according to their degree centrality in the network. The degree-centrality is computed as the total number of inbound and outbound links connected to a router. The optimal number of clusters are obtained by observing the network performance (in terms of cache hit ratio) for different number of clusters. Therefore, the network clustering is dynamic and changes for different network topologies. These “k” routers are designated as the initial centroids $\left(Centroid_{i}\in C_{i}\right)$ before start clustering of the network nodes. Using degree centrality metrics, the clusters would be tightly coupled as more number of routers become adjacent to the centroids. It is mentioned in step-1 and step-2 of Algorithm 1. It would also be interesting to analyze the other metrics for selection of initial centroids such as betweeness centrality and closeness centrality. However, the earlier works [15,44] in this direction have shown that the node degree centrality is a sufficiently good criteria for network clustering. Additionally, the time complexity to determine the degree centrality in a network topology is $O(V^{2})$, which is much lesser than the time complexity to compute betweeness and closeness centrality measures that have the time complexity of $O(VE+V^{2})$. Therefore, the degree centrality measure is used to select initial centroids.
**Algorithm 1:** Proposed network clustering scheme**Input:** All the network routers $R_{j}$, where j=1,2,…,|R|.**Output:** Set of “k” clusters $\left(C_{i}\right)$, where i=1,2,…,k.
Sort the routers according to their decreasing order of degree centrality.Designate top “k” routers as initial centroids that have higher degree centrality $\left(Centroid_{i}\in C_{i}\right)$.Iterate *step*-3(*a*), 3(*b*) and *step*-4, till there is a change in centroids:(a)Determine the distance between the routers $\left(R_{j}\right)$ and each of the centroid $\left(Centroid_{i}\right)$ using following equation:
(1)$$Dist\left(Centroid_{i},R_{j}\right)=\frac{H(Centroid_{i},R_{j})}{Min(B\left(Centroid_{i},R_{j}\right))}$$(b)Assign each router $\left(R_{j}\right)$ to the closest centroid $\left(Centroid_{i}\right)$, i.e., $R_{j}\in C_{i}$.Determine the new centroid $\left(Centroid_{i}\right)$ in each cluster that has minimum distance from the intra-cluster routers.

Then, the scheme determines the distance of each router $\left(R_{j}\right)$ from all the centroids $\left(Centroid_{i};\;i\in \left\{1,2,\ldots ,k\right\}\right)$ as illustrated in step-3(*a*). The distance between a centroid $Centroid_{i}$ and the router $R_{j}$ is determined using the hop count and bandwidth parameters as defined in Equation (1). The probability to associate a router into a specific cluster increases with a decrease in the number of hops between its centroid and the router. The value of distance parameter $\left(Dist\left(Centroid_{i},R_{j}\right)\right)$ decreases with an increase in the bandwidth between the centroid and the router. Therefore, using Equation (1), the router is assigned to a centroid that has minimum hop count from the router and is also connected through the high bandwidth links to form tightly coupled clusters (shown in *step*-3(*b*)). It improves the efficiency of content forwarding from one node to another node within the clusters using higher bandwidth connections. After each iteration of the cluster formations, the router that has minimum distance (computed using Equation (1)) from its intra-cluster routers is designated as a new centroid for its cluster. If the centroids are changed as compared to the previous iteration, then step-3 is executed again. Otherwise, if there is no change in centroids, then it indicates that the cluster formation process is completed and the routers are partitioned into “k” clusters. After clustering of the network routers, the end-user devices connected with the edge routers also become part of their respective clusters.

### 5.2. Structure of Interest and Content Message

The proposed caching scheme considers the cluster information, content popularity, and hop count parameters for caching decisions. Therefore, the structures of Interest and Content messages are updated to store information for these parameters.

Towards this, each Interest message $I_{j}$ is updated with the novel fields, $H(I_{j})$ and $Clus(I_{j})$ as shown below.



**Structure of Interest message:**


$Name(I_{j})$



$H(I_{j})$



$Clus(I_{j})$

…


Here, the name of the requested content is stored in the $Name(I_{j})$ field. The $H(I_{j})$ field stores the total number of hops traversed by the Interest message $\left(I_{j}\right)$. The $Clus(I_{j})$ field contains the unique identification number of the cluster in which the $I_{j}$ is generated by the end-user device $\left(U_{u}\right)$ in the network. This unique cluster identification id is identical for all the end-user devices and routers that are grouped together in a cluster and unique for different clusters.

As the content caching operations are performed during the Content (Data) message $\left(D_{j}\right)$ forwarding towards the end-user devices, the $H(I_{j})$, $Clus(I_{j})$ and $H(D_{j})$ fields are appended in $D_{j}$ for efficient caching decisions. The structure of the content message is illustrated below.



**Structure of Content message:**


$Name(D_{j})$



$H(I_{j})$



$Clus(I_{j})$



$H(D_{j})$



$Clus(D_{j})$



η

…


The name of the requested content is stored in the $Name(D_{j})$ field. The $H(I_{j})$ field contains the hop count information which is traversed by the Interest message $\left(I_{j}\right)$ from the end-user device to reach the content provider. The value of $H(I_{j})$ and $Clus(I_{j})$ field in the $D_{j}$ are replicated from the Interest message $\left(I_{j}\right)$ to $D_{j}$ and the count of hops traversed by $D_{j}$ is stored in the field $H(D_{j})$.

### 5.3. Interest Message Forwarding Mechanism

In this section, the Interest message forwarding and processing mechanism are discussed and summarized in Algorithm 2 (Interest message forwarding mechanism). As shown in step-1 of the algorithm, when an end-user device $\left(U_{u}\right)$ requires a content (Data) $D_{j}$, then it prepares the corresponding Interest message $I_{j}$ with the requested content name as $Name(I_{j})$ and initializes the $HC(I_{j})$ field as 0. The network is already clustered according to the proposed clustering scheme and each cluster has a unique identification number which is same for all the intra-cluster nodes (end-users and routers). Therefore, the device $U_{u}$ write its cluster identification id in the $Clus(I_{j})$ field of $I_{j}$ and forwards it to the adjacent router $R_{i}$(step)-2). On receiving the message $I_{j}$, each on-path router $R_{i}$ increases the value of $H(I_{j})$ field by 1 (step-3(*a*)) and insert the requested content name $Name(I_{j})$ in its Popularity Table according to FIFO replacement mechanism as shown in step-3(*b*). Then, $R_{i}$ searches its cache for the requested content and if the content exists then Algorithm 3 (Content message forwarding and caching mechanism) (discussed in Section 5.4) is executed. Otherwise, the traditional Interest message forwarding process is executed as illustrated in step-3(*d*) to (*f*) and elaborated in Section 2.
**Algorithm 2:** Interest message forwarding mechanism $\left(U_{u},I_{j},R_{i},R_{m}\right)$$U_{u}$ prepares an Interest message $\left(I_{j}\right)$ to retrieve the content $D_{j}$ and initialize $H(I_{j})=0$.$U_{u}$ writes its unique cluster identification id in the $Clus(I_{j})$ field of $I_{j}$ and forward towards its adjacent upstream router $R_{i}$.Then, any intermediate router $R_{i}$ performs following steps after receiving $I_{j}$.(a)Update the value of $H(I_{j})$ field as $H\left(I_{j}\right)=H\left(I_{j}\right)+1$.(b)If $Max(|PT_{R_{i}}|)$ > $|PT_{R_{i}}|$, then insert $Name(I_{j})$ in $PT_{R_{i}}^{s}$, where “s” represents the next empty slot in the Popularity Table of $R_{i}$. Else, if $Max(|PT_{R_{i}}|)$=$|PT_{R_{i}}|$, then insert $Name(I_{j})$ in $PT_{R_{i}}^{s}$ using FIFO replacement mechanism.(c)If requested content exists in the $CS(R_{i})$ then navigate to *Algorithm 3: Content message forwarding and caching mechanism*.(d)Else, if PIT of $R_{i}$ has a record for $I_{j}$, then aggregate $I_{j}$ in its PIT.(e)Else, Search the FIB of $R_{i}$ to forward $I_{j}$ to appropriate upstream router. If entry found, then forward $I_{j}$ accordingly and create an entry in the PIT.(f)Else, discard $I_{j}$ from the network.

**Algorithm 3:** Content message forwarding and caching mechanism $\left(U_{u},D_{j},R_{m}/serv,R_{y}\right)$
If requested content exists in the $CS(R_{m})$ or $I_{j}$ reaches the server (serv), then following steps are performed:(a)Prepare a Content message $D_{j}$ with initializing corresponding field $Name(D_{j})$ and the requested payload.(b)Replicate the values of $Clus(I_{j})$ and $H(I_{j})$ fields from $I_{j}$ to the $Clus(I_{j})$ and $H(I_{j})$ fields of $D_{j}$.(c)Initialize, $H(D_{j})=0$.(d)The content provider $\left(R_{m}/serv\right)$ writes its unique cluster identification id $(Clus\left(R_{m}\right)/Clus\left(Serv\right)$ in the $Clus(D_{j})$ field of $D_{j}$.(e)Initialize the boolean field η as TRUE.(f)Transmit $D_{j}$ towards $U_{u}$.When $D_{j}$ reaches to an intermediate router $R_{y}$, then $R_{y}$ perform following steps for caching decisions and content forwarding towards $U_{u}$.Update the value in $H(D_{j})$ field as $H(D_{j})$ = $H(D_{j})+1$.If $Clus\left(I_{j}\right)\neq Clus\left(R_{y}\right)$ or $Clus\left(I_{j}\right)=Clus\left(D_{j}\right)$, then move to step-6.Else,(a)Compute, $PT_{R_{y}}(Name\left(D_{j}\right)$ in $PT_{R_{y}}$.(b)Compute, $Caching\_Gain=PT_{R_{y}}\left(Name\left(D_{j}\right)\right)\times \frac{H(D_{j})}{H(I_{j})}$(c)If $T_{R}\leq Caching\_Gain$ and η=TRUE then,
Cache $D_{j}$ in the $CS(R_{y})$ using LFU cache replacement strategy.Reset η=FALSE.$R_{y}$ forwards $D_{j}$ towards the $U_{u}$ using its PIT.


### 5.4. Content Message Forwarding and Caching Mechanism

This section elaborates Content message forwarding and caching mechanism which is summarized in Algorithm 3: (Content message forwarding and caching mechanism). When requested content is found in the cache of router $R_{m}$ or the Interest message $I_{j}$ reaches the server (serv), then $R_{m}/serv$ prepares a Content message $D_{j}$ with the requested payload as shown in step-1 of Algorithm 3. Then, the content provider $\left(R_{m}/serv\right)$ replicates the values of $Clus(I_{j})$ and $H(I_{j})$ fields from $I_{j}$ to corresponding fields of $D_{j}$ and reset the value of $H(D_{j})$ to 0. Subsequently, the $\left(R_{m}/serv\right)$ write its unique cluster identification id in the $Clus(D_{j})$ field of $D_{j}$ and set the value of boolean variable (η) to “TRUE” which indicate that the caching is enabled for the content in the on-path routers step-1(*d*) to 1(*e*). The content provider then forward the message towards its requester $\left(U_{u}\right)$. In the path, the intermediate router $R_{y}$ perform step-2 to 6 for content caching and forwarding operations. As illustrated in step-3, the on-path router $R_{y}$ increases the hop count value of $H(D_{j})$ field by 1.

In the proposed caching scheme, at most one copy of the content is cached in those routers $\left(R_{y}\right)$ which belong to the cluster that has generated the request $(\left(Clus\left(I_{j}\right)=Clus\left(R_{y}\right)\right)$. The routers that belong to other intermediate clusters perform content forwarding operations without its caching. This approach minimizes computational and caching delay as shown in step-4. Moreover, to reduce cache replacements and content redundancy, the content is not cached in the intermediate routers if the content provider $\left(R_{m}/serv\right)$ and the requester $\left(U_{u}\right)$ exists in the same cluster $\left(Clus\left(D_{j}\right)=Clus\left(I_{j}\right)\right)$ as shown in *step*-4. Otherwise, if the Interest message is generated from the different cluster than the content provider then, following steps are performed. For caching decisions in $R_{y}\;\left(Clus\left(R_{y}\right)=Clus\left(I_{j}\right)\right)$, the popularity of $D_{j}$ is determined by counting the occurrences of requests for $D_{j}$ in the $PT_{R_{y}}$ as mentioned in step-5(*a*). Then, the Caching_Gain is computed as the product of content popularity and the normalized hop count parameter (step-5(*b*)). The normalized hop count is determined as the ratio of $H(D_{j})$ and $H(I_{j})$. According to step-5(*b*), the Caching_Gain increases with an increase in the content popularity and the distance traversed by the content message $D_{j}$. Therefore, the popular contents are placed near the edges of the network with a higher probability, and the excessive content redundancy is controlled using the proposed clustering-based mechanism. Once the cache of the intermediate router is full, the LFU replacement algorithm is used to substitute the least popular content with the incoming content that has $Caching\_Gain\geq T_{R}\left(Threshold\right)$. The content caching operation is performed only when the value of η is “TRUE” which indicate that the content $\left(D_{j}\right)$ is not cached in the cluster $\left(Clus\left(R_{y}\right)\right)$. To ensure that at most one router cache the incoming content $\left(D_{j}\right)$ in the requester’s cluster, the value η is reset to “FALSE” after content caching. Finally, each intermediate router $\left(R_{y}\right)$ forwards the Content message towards the requester $\left(U_{u}\right)$, irrespective of the caching decision as defined in step-5.

### 5.5. An Illustration of Proposed Content Message Forwarding and Caching Mechanism

As discussed in Section 4, suppose the network is partitioned into three different clusters as shown in Figure 1 and an Interest message for “\prefix\xyz” (represented as $I_{i}$ now onwards) is generated by $U_{3}$ and forwarded in the network through the route: $U_{3}\to R_{1}\to R_{2}\to R_{5}\to R_{7}\to Serv$. It has also been shown in Section 4 that in the proposed caching scheme, the content caching decisions are taken by $R_{1}$ and $R_{2}$ based on the content popularity and hop count parameters as the request has been generated from Cluster $C_{1}$. Suppose the size of the Popularity Table is 10 in $R_{1}$ and $R_{2}$ and the count of Interest messages for $I_{i}$ in the Popularity Table are 5 $\left(PT_{R_{1}}\left(Name\left(D_{i}\right)\right)\right)$ and 6 $\left(PT_{R_{2}}\left(Name\left(D_{i}\right)\right)\right)$, respectively. As the requested content is fetched from the server, the value of $H(I_{i})$ would be 5. The value of $H(D_{i})$ would be 4 and 3 at router $R_{1}$ and $R_{2}$, respectively. Then, the Caching_Gain would be computed for router $R_{2}$ using step-5(*b*) of Algorithm 3 as follows:

(2) $$Caching\_Gain=PT_{R_{2}}\left(Name\left(D_{i}\right)\right)\times \frac{H(D_{i})}{H(I_{i})}=6\times \frac{3}{5}=0.36$$

Suppose, the value of $T_{R}$ is 0.4, then according to step-5(*c*), the content would not be cached in $R_{2}$ because $\left(T_{R}>Caching\_Gain\right)$. Then, the content message $D_{j}$ would be forwarded towards $R_{1}$ with η=TRUE. On receiving $D_{j}$, $R_{1}$ would compute the Caching_Gain as follows:

(3) $$Caching\_Gain=PT_{R_{1}}\left(Name\left(D_{i}\right)\right)\times \frac{H(D_{i})}{H(I_{i})}=5\times \frac{4}{5}=0.4$$

In this case, the value of $T_{R}\leq Caching\_Gain$. Therefore, the content would be placed in the cache of $R_{1}$ and then it would be forwarded to end-user device $U_{3}$.

On the other side, if the content is cached in $R_{2}$ after computation of Caching_Gain, then the value of η become FALSE and the router $R_{1}$ does not cache the content. Therefore, the proposed caching scheme ensures that at most one copy of the incoming content message is cached in the routers of requesting cluster to increase content diversity in the network.

As the proposed scheme does not consider the router’s importance (such as degree centrality, betweeness centrality etc.) during content placement decisions, the network load is not concentrated on a few network routers. Moreover, the proposed caching scheme does not require cluster heads for Interest/Content message forwarding and caching operations. Thus, the network traffic and computations are distributed among the network routers and the scheme does not suffer from the load balancing and bottleneck issues.

## 6. Performance Evaluation

This section first discusses the simulation environment and the values of its parameters. After this, the performance of the proposed caching scheme is evaluated in terms of the cache hit ratio, average network hop count, delay, and network traffic metrics. Then, the obtained results are compared with the peer caching schemes such as traditional caching strategy (LCE) [27], DC-based [15], FGPC [13], and recently proposed CPNDD [17] and PDC [34] schemes.

### 6.1. Simulation Environment

The ndnSIM simulation tool [45] is used to examine the performance of the proposed and the peer caching schemes in the CCN environment. For simulation setup, a network topology is build based on the Abilene network [46]. The Abilene network topology is implemented in the United States for connectivity among the academic institutions, Universities and other affiliated organizations across the District of Columbia and Puerto Rico. The performance of most of the existing and recent caching schemes have also been examined on the Abilene network topology such as DC-scheme [15], PDC [34] and CPNDD [17] strategies. Therefore, this topology is used for performance evaluation of the caching solutions. The network topology connects the nodes using up to 10 Mbps (bandwidth of network connections ranges between 1 and 10 Mbps) connections having a link delay of 10 ms. It contains 167 nodes which comprise 133 end-user devices (requesters), 33 routers, and 1 content server. The topology has 11 core routers and 22 edge routers. The edge routers are directly connected with the end-user devices and each end-user is connected with just one of the edge routers.

The server (serv) stores 5000 contents altogether that can be requested in the network and hence, the content catalogue size |Ctlg| is 5000. The payload size of each content message is 1 KB. The cache size of in-network routers is set to 1% $\left(|CS\left(R_{i}\right)|=50\right)$ and 2% $\left(|CS\left(R_{i}\right)|=100\right)$ of the content catalogue size to obtain realistic results under different simulation configurations. The content access pattern follows Zipf distribution with skewness parameter α=0.7 [34]. The Interest message generation frequency (λ) is 50/s for each end-user device and nearly 1 million content requests are generated in 1000 STU (Simulation Time Unit) during performance evaluation of the content caching strategies. One of our prior work [34] suggested that the size of the Popularity Table is directly proportional to content catalog size. Hence, for reliable and accurate determination of the content popularities, the size of Popularity Table is set to 1% of the content catalog for effective content caching decisions, which is $(Max(|PT_{R_{i}}\left|\right)$ = 0.01×|Ctlg|=500) for each router. It has also been observed that increasing the size of Popularity Table beyond this value, does not increase the QoS for requesters in a linear manner and increases the computational overhead in the network routers. Therefore, the Popularity Table is implemented with 500 slots in each network router to determine the content access frequencies reliably.

**Figure 3:**
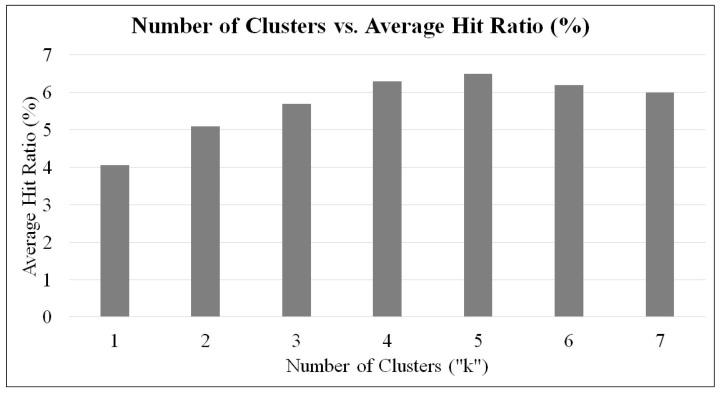
Average hit ratio on different number of clusters in Abilene network topology with |Ctlg|=5000, $|CS(R_{i})|=50$, α=0.7, λ=0.7, $|PT_{R_{i}}|=500$.

Before performance evaluations, the Abilene network topology is clustered into different number of non-overlapping clusters (k={1,2,3,…,7}) using the proposed clustering mechanism. To determine the appropriate number of clusters (“*k*”), the cache hit ratio has been computed with |Ctlg|=5000, $|CS(R_{i})|=50$, α=0.7, λ=0.7, $|PT_{R_{i}}|=500$ on k={1,2,3,…,7}. The average cache hit ratio (%) obtained for different number of clusters is illustrated in Figure 3. As shown in Figure 3, the optimal cache hit ratio is achieved when k=5, and thus, the network is partitioned into 5 clusters.

To determine the optimal threshold value $\left(T_{R}\right)$ for caching decisions, the simulation executions are performed for different values of threshold ranging between $\left(T_{R}=\left\{0.1-10.0\right\}\right)$ with above mentioned network configurations. The average network delay metric is used to examine the optimal value of $T_{R}$ and the minimum value of this metric is achieved with $T_{R}=1.5$. Hence, this value is used during the comparison of the proposed caching scheme with peer strategies. Although the threshold value and the number of clusters have been selected based on the empirical study on a standard network topology and may change for other CCN topologies, it provides a good foundation to evaluate the performance of the proposed caching scheme on large-scale CCN-enabled networks.

### 6.2. Performance Evaluation of Caching Schemes: Cache Hit Ratio (%)

A cache hit occurs when the incoming Interest message is satisfied using the cached copy from the network routers. Contrarily, if the requested content is not found in the CS of the router, then the cache miss happens. The network cache hit ratio (%) is the percentage ratio of the number of cache hits and the total number of Interest messages received by all the routers in the network. The increase in the cache hit ratio decreases the content retrieval delay and the load from servers. The cache hit ratio represents the effectiveness of caching scheme to reduce the redundant traffic in the network. The gain in cache hit ratio is computed as the difference between the average cache hit ratio achieved by the proposed scheme and the existing caching schemes.

**Figure 4:**
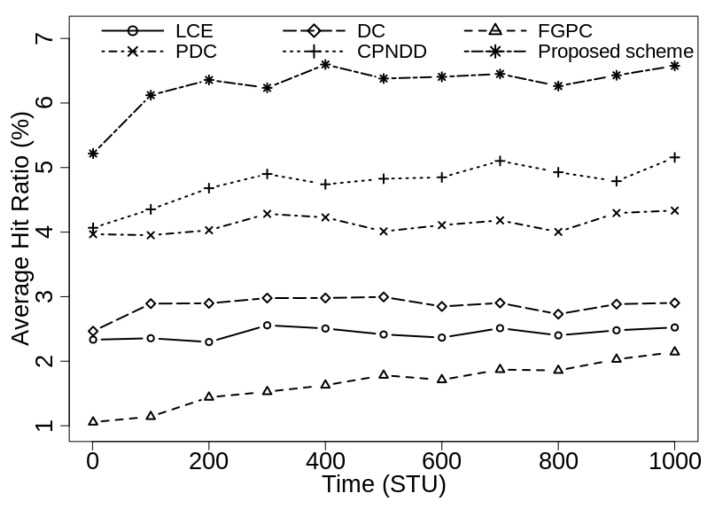
Comparison of cache hit-ratio (%) with λ=50/s, $|CS(R_{i})|=50$, α=0.7, and |Ctlg|=5000.

Figure 4 shows the average hit ratio obtained by various caching schemes when caching capacity of in-network routers is 50 (1% of Ctlg). In the beginning, the cache hit ratio of all the schemes is low because the in-network cache are initially empty and the required contents are retrieved from the server. In this scenario, the traditional LCE caching scheme, FGPC, and DC-based schemes show poor hit ratio due to their underlying heuristics and the proposed scheme outperforms them by achieving up to 4.1%, 4.5%, and 3.7% gain from them, respectively. The proposed scheme also shows up to 1.5% and 2.3% gain from recently proposed CPNDD and PDC caching strategies, respectively.

**Figure 5:**
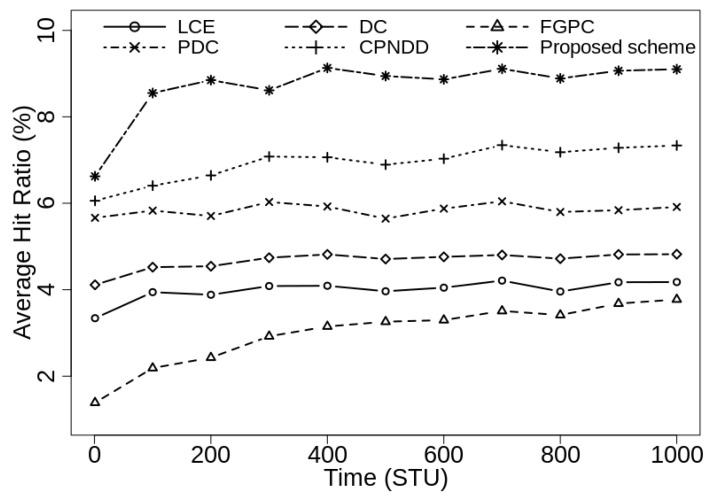
Comparison of cache hit-ratio (%) with λ=50/s, $|CS(R_{i})|=100$, α=0.7, and |Ctlg|=5000.

Figure 5 illustrates the average cache hit ratio when the caching capacity of network routers increases to 100 (2% of |Ctlg|). In this case, the proposed and existing caching schemes shows significant improvement in the cache hit ratio from the previous simulation scenario where $\left|CS(R_{i})\right|$ was 50. With larger caching capacity, the proposed scheme shows up to 5.0%, 4.3%, 5.4%, 3.2%, and 1.8% gain in hit ratio from the LCE, DC-based, FGPC, PDC, and CPNDD caching schemes, respectively. This gain is achieved as the proposed clustering-based caching scheme places popular contents near the edge routers with reduced intra-cluster content redundancy and more space is allocated for the content caching. Thus, the available cache space is fairly used by popular contents in the network.

### 6.3. Performance Evaluation of Caching Schemes: Average Hops Count to Retrieve Requested Content

The number of hops traversed by the Interest message for cache hit (or the number of hops between the end-user and the server, in the case of a cache miss on all intermediate routers) is defined as the hop-count to retrieve the requested content. The average hop count is computed as the average number of hops that are traversed to satisfy the Interest messages in the network. It is desired that the value of the average hop count should be smaller for improved QoS for end-user devices. The percentage of hop count reduction is computed using Equation (4) as mentioned below. Here, %H_reduc, H(E.S.) and H(P.S.) represent the percentage of reduction in hop count, the number of hops observed under the existing caching scheme, and hop count experienced in the proposed caching scheme, respectively.

(4) $$\%H\_reduc=\frac{(H(E.S.)-H(P.S.))\times 100}{H(E.S.)}$$

**Figure 6:**
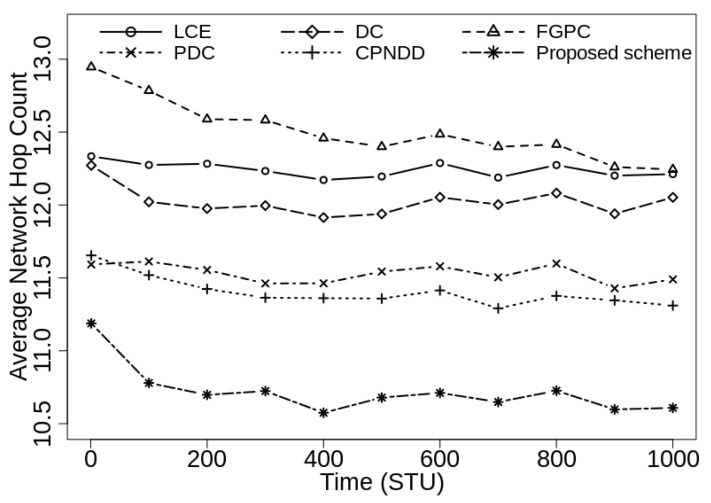
Comparison of average network hop count with λ=50/s, $|CS(R_{i})|=50$, α=0.7, and |Ctlg|=5000.

Figure 6 shows the average network hop count observed in the proposed and peer caching schemes under identical simulation conditions with $|CS(R_{i})|=50$. As the proposed scheme places popular contents in the routers and evicts less-popular contents during cache replacement decisions, more requests are served by the intermediate routers than the server. Hence, the content retrieval path is shortened and the QoS for the end-user devices improves. During simulations, the proposed caching scheme reduces the average network hop count up to 13.2%, 12.0%, 13.4%, 7.7%, and 6.2% from the LCE, DC-based, FGPC, PDC, and CPNDD caching strategies, respectively.

**Figure 7:**
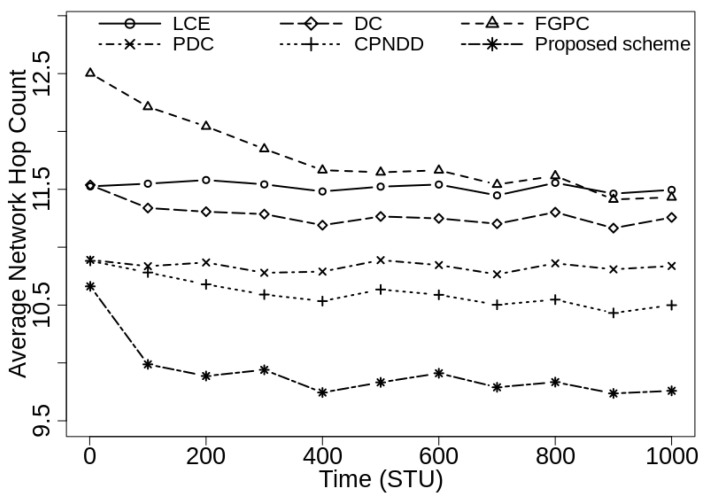
Comparison of average network hop count with λ=50/s, $|CS(R_{i})|=100$, α=0.7, and |Ctlg|=5000.

Figure 7 shows the average network hop count experienced by the end-user devices when caching capacity of in-network routers is increased to 100 contents with keeping other simulation parameters remain unchanged. During executions, similar to previous results, the proposed scheme shows a 7.1–15.1% reduction in average hop count metric from the peer caching schemes. These results prove that the proposed strategy effectively reduces the number of hops in retrieving the required content as compared to other schemes.

### 6.4. Performance Evaluation of Caching Schemes: Average Network Delay (in Microseconds)

The average network delay is determined as the total time (in microseconds) between preparing the Interest message and receiving the requested content. It also includes the request retransmission delay, if the content is not received within the defined duration. This metric represents the performance of the network from the perspective of end-user devices. The reduction in average network delay signifies improved network performance as the content is retrieved from the nearby routers.

**Figure 8:**
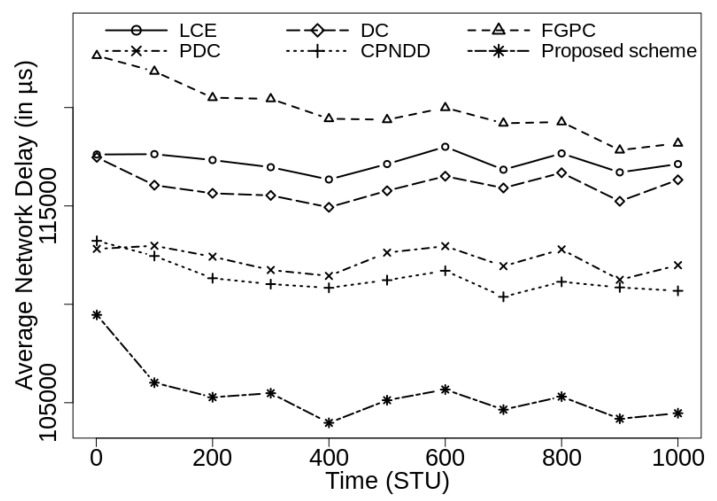
Comparison of average network delay (in μs) with λ=50/s, $|CS(R_{i})|=50$, α=0.7, and |Ctlg|=5000.

**Figure 9:**
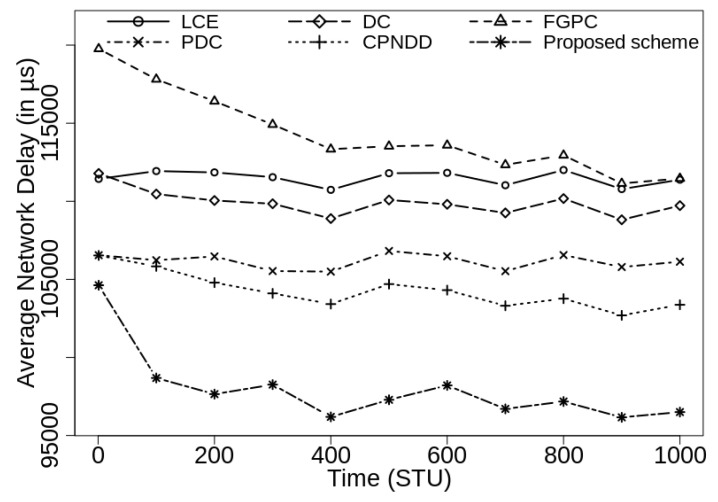
Comparison of average network delay (in μs) with λ=50/s, $|CS(R_{i})|=100$, α=0.7, and |Ctlg|=5000.

Figure 8 and Figure 9 show the average network delay observed under different caching schemes for the caching capacities of 50 and 100, respectively. As expected, the proposed caching scheme shows the least average network delay as it focuses on caching the popular contents near the edges of the network with reduced content duplications.

### 6.5. Performance Evaluation of Caching Schemes: Average Network Traffic (in KB/s)

The average network traffic is computed as the total amount of data on network connections in per unit time and represented in terms of KB/s. This metric is used to examine the efficiency of the caching schemes and content transmissions in the network. The proposed clustering-based caching scheme does not flood the Interest messages in the network and supports efficient caching decisions using the network clusters, content popularity and distance parameters. Therefore, the network traffic is reduced for identical content transmissions and more diverse contents are accessed from the nearby devices. The percentage reduction in average network traffic is determined using Equation (5). In this equation, the variables %T_reduc, T(P.S.), and T(E.S.) define the percentage reduction in average network traffic, and average network traffic observed under proposed scheme and existing peer scheme, respectively.

(5) $$\%T\_reduc=\frac{(T(E.S.)-T(P.S.))\times 100}{T(E.S.)}$$

**Figure 10:**
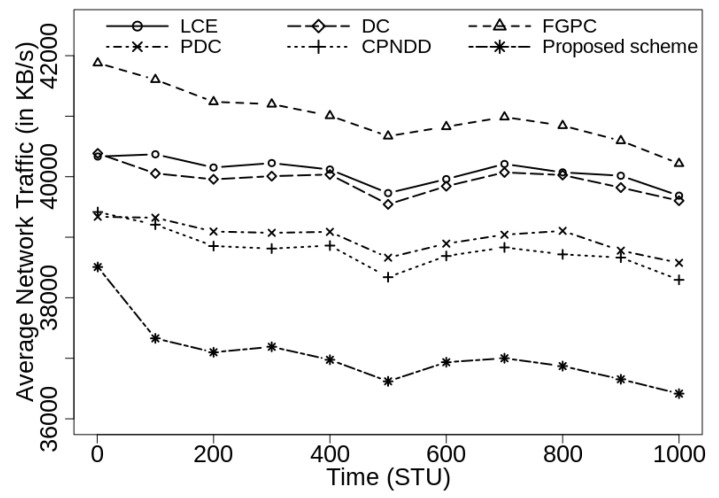
Comparison of average network traffic (in KB/s) with λ=50/s, $|CS(R_{i})|=50$, α=0.7, and |Ctlg|=5000.

Figure 10 shows the simulation results for average network traffic with $|CS(R_{i})|=50$. The results display how the proposed caching mechanism effectively reduces the traffic and load from the network connections. In this scenario, the proposed caching scheme shows up to 8.3%, 8.1%, 9.5%, 5.6%, and 4.9% reduction in the network traffic from the competing LCE, DC-based, FGPC, PDC, and CPNDD caching schemes, respectively.

**Figure 11:**
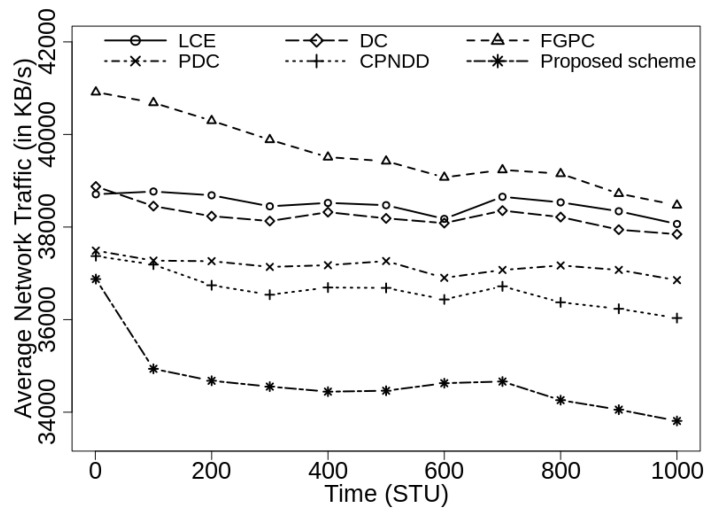
Comparison of average network traffic (in KB/s) with λ=50/s, $|CS(R_{i})|=100$, α=0.7, and |Ctlg|=5000.

It has also been observed that a direct correlation exists between the average traffic and the average network delay metrics. The smaller average network delay implies that the requested contents are found near the end-user devices and thus, a lesser number of hops are traversed to retrieve the content. This leads to decreased network traffic and increases the use of network resources. As the $\left|CS(R_{i})\right|$ increases to 100, the average network traffic reduces for all the caching schemes because more contents are cached in the intermediate routers. In this scenario also, the proposed caching scheme outperforms the existing strategies by achieving up to 11.2% reduction in the average network traffic from LCE and peer caching strategies as shown in Figure 11.

## 7. Conclusions

This paper starts with presenting various existing content placement schemes for the CCN environment in the literature. Then, a novel network clustering-based content caching scheme is proposed in which the intra-cluster routers cooperate with each other during content placement decisions. The proposed scheme considers the cluster information, content popularity, and hop count parameters to effectively use the available cache resources. In the proposed strategy, the network routers are clustered based on the joint consideration of hop count and the bandwidth parameters. Using the network clustering mechanism, the excessive cache replacement operations and the computational latency reduces significantly without additional communication overhead. Using proposed caching heuristics, the scheme increases the probability to cache the popular contents close to the end-user devices. Finally, the widespread simulations are performed with realistic network configurations and the performance of the proposed caching scheme is examined on cache hit ratio, average network hop count, network delay, and traffic metrics. The results showed that the proposed scheme outperforms the traditional CCN caching scheme along with peer heuristic-based DC-based, FGPC, PDC, and CPNDD caching strategies.

In future works, the performance of the proposed strategy will be analyzed in mobility-based networks and the recent network topologies such as Geant, Tiger2, DTelekom and Internet2 etc. Additionally, more parameters can be integrated with the existing solution for further improvement in network performance.

## Figures and Tables

**Table 1:** Features of the existing caching schemes.

Scheme/Author	Content Placement Attribute(s)	Content Replacement Attribute(s)	Hit-Ratio	Content Popularity Table	Network Clustering	Network Delay	Network Traffic	Content Redundancy
LCE [27]	Cache everywhere	LRU, LFU	Low	NA	No	High	High	Very high
RandProb [28]	Random allocation	LRU	Low	No	No	High	High	Moderate
LCD [29]	Immediate downstream node	LRU	Low	No	No	High	High	Moderate
Probcache [18]	Hop count, caching capacity	LRU	Moderate	No	Yes	Moderate	Moderate	High
Chai et al. [31]	Betweeness centrality	LRU	Low	No	No	High	High	High
DC-based [15]	Node degree	LRU	Moderate	No	No	Moderate	High	Moderate
CPNDD [17]	Node degree and hop count	LRU	Moderate	No	Yes	Moderate	Moderate	Moderate
MPC [32]	Content popularity	LRU	Moderate	No	Yes	Moderate	High	High
FGPC [13]	Content popularity	LRU	Moderate	Yes	No	Moderate	Moderate	High
CPUL [33]	Content popularity and user location	LRU, LFU	Low	No	Yes	High	High	Moderate
DPWCS [14]	Content popularity	LRU	Moderate	Yes	No	Moderate	Moderate	High
PDC [34]	Content popularity and hop count	LRU	Moderate	Yes	No	Moderate	Moderate	High
HCC [35]	Node degree and hop count	LRU	Moderate	No	Hierarchical	High	High	Moderate
Sourlas et al. [36]	Hash-based	LRU	Moderate	No	k-split, k-medoid	Moderate	Moderate	Low
Hasan et al. [37]	Content popularity	LRU	Low	Yes	Clique	High	High	Moderate

**Table 2:** Variables Notation.

Variable	Definition
$CS(R_{i})$	Content Store of router $R_{i}$
** serv **	**Set of content servers in the network**
$\left|CS(R_{i})\right|$	Content caching capacity of $R_{i}$
$PT_{R_{i}}^{s}$	*s*th slot in the Popularity table of $R_{i}$.
$|PT_{R_{i}}|$	Number of occupied Popularity Table slots in $R_{i}$
$Max(|PT_{R_{i}}|)$	Maximum size of Popularity Table for $R_{i}$
$PT_{R_{i}}^{s}$	*s*th slot of Popularity Table in $R_{i}$
$I_{i}$	Interest message with requested content name $Name(I_{i})$.
$D_{i}$	Content message corresponding to Interest message $I_{i}$ with requested content name (i).
$PT_{R_{i}}(Name\left(D_{j}\right)$	Number of occurrences of content name $(Name\left(D_{j}\right)$ in $PT_{R_{i}}$.
λ	Request rate from each end-user device in per unit time
$H(I_{j})$	Number of in-network routers and servers traversed by the message $I_{j}$.
$H(D_{j})$	Number of in-network routers and servers traversed by the Content message $D_{j}$.
$H(R_{i},R_{j})$	Number of in-network routers between the routers $R_{i}$ and $R_{j}$.
$Min(B\left(R_{i},R_{j}\right))$	Minimum bandwidth in the intermediate links between $R_{i}$ and $R_{j}$.
α	Exponent value in Zipf distribution
$Clus(I_{j})$	Unique identification number of the cluster in which $I_{j}$ is generated.
$Clus(R_{i})$	Cluster Identification number in which $R_{i}$ resides.
η	Boolean variable to control intra-cluster caching operations.
$T_{R}$	Threshold value for caching decisions in the network routers.
|Ctlg|	Number of distinct contents in the network

## Data Availability

Not applicable.
